# A phenomenological analysis of relapse among adults with substance abuse

**DOI:** 10.1038/s41598-026-39291-z

**Published:** 2026-02-11

**Authors:** Anemut Mehari, Henok Melese, Mohammed Reshid

**Affiliations:** 1 School of Psychology, Addis Ababa University, Addis Ababa, Ethiopia; 2 Department of Psychology, Dilla University, Dilla, Ethiopia; 3 Department of Psychology, Woldia University, Woldia, Ethiopia

**Keywords:** Substance use relapse, Adults, Phenomenology, Coping perspectives, Psychology, Health occupations

## Abstract

Relapses from substance usage continue to be a major global public health concern. Many individuals find it difficult to sustain long-term recovery, often cycling between phases of recovery and relapse, even with improvements in treatments. This study employs a center-based phenomenological design to examine the experiences of adults who relapse from substance dependence. For in-depth interviews, nine patients were chosen. An interpretative phenomenological analysis (IPA) was used to analyze the data. The results revealed that a complex interplay of personal and environmental factors influences relapse. Self-motivation, relocating, maintaining supportive social networks, improving mental resilience, making plans for future stability, and actively engaging in spiritual healing were all effective coping strategies for reducing relapse. To prevent recurrence and encourage long-term recovery, the findings emphasize the need for comprehensive intervention strategies that address social influences, environmental triggers, and emotional regulation.

## Introduction

Substance use, including alcohol, tobacco, khat, and cannabis typically begins during adolescence and is most prevalent among young people. Adolescents and young adults (aged 12–24) report higher rates of use than older age groups, with 83% consuming alcohol and 33% using cannabis^1,2^. Initiation and continued use are commonly influenced by peer pressure, media exposure, inadequate guidance, poor anger management, easy drug accessibility, and affiliation with substance-using peers^3,4^.

Relapse - a central concern in substance use recovery is commonly defined as the return to previous or problematic patterns of use following a period of abstinence^5,6^. Global research consistently shows that relapse is widespread, with rates exceeding 50% within the first year of treatment^7–11^. In Ethiopia, substance use is also most common among young adults, with alcohol and khat being the most frequently reported substances^4,12^. Prevalence estimates vary widely, ranging from 14.1% ^13^ to 62.4% among university students^14,15^. Treatment facilities similarly report that nearly half of admitted patients fall between the ages of 21 and 28, underscoring a youth-centered burden^3^. Relapse, however, remains underexplored, although Abegaz reported a 48% relapse rate that was broadly consistent with global patterns (e.g., 50.29%)^11^.

Despite substantial evidence on substance use as a public health concern in Ethiopia, limited qualitative research has explored adults’ lived experiences of relapse, including the psychosocial and environmental influences involved. National survey data indicate that nearly half of Ethiopian youth aged 15–24 reported using at least one substance such as alcohol, khat, or tobacco within 30 days of the survey, with alcohol and khat particularly common in both urban and rural areas^16^. Community-based studies also show high lifetime and current prevalence of substance use among young people, with significant proportions reporting alcohol, khat, tobacco, and cannabis use and varying patterns across regions and populations^17^. Although quantitative studies have identified relapse-associated factors such as khat chewing and stressful life situations among clinical subgroups in Ethiopia^18^, little is known about how adults experience relapse, what psychosocial and environmental factors contribute to relapse, and which coping strategies are effective for sustained recovery in the Ethiopian context. Understanding relapse also requires attention to psychological and social challenges, including stigma, stress, depression, and familial expectations^19,20^. Therefore, this phenomenological study investigates the personal experiences of relapse, the biopsychosocial factors contributing to substance use relapse, and the coping strategies, drawing on both theoretical and empirical perspectives^21–27^, employed by adults in Ethiopia. This approach provides context-specific qualitative insights to inform more effective recovery interventions. It also evaluates strategies to mitigate relapse risk, drawing on existing evidence highlighting the chronic and multifaceted nature of substance use relapse disorders. Based on these gaps, the study addresses the following research questions:

1. How do adults in Ethiopia experience relapse?
2. What factors influence relapse among adults in Ethiopia?
3. What coping strategies are effective for adults experiencing relapse in Ethiopia?

## Methods

### Research setting

Amanuel Mental Health Specialized Hospital was the first and oldest hospital established in 1930 for helping individuals with mental health and substance abuse problems. It is located in western Addis Ababa. In its in-and-out patient service program, nearly 26,400 follow-up and new mental health patients receive services annually^28^. One of the main problems at the center is substance usage^28^. The relapse rate is also higher than that in other addiction treatment facilities^3^, with 12% experiencing one-time relapse, 32% experiencing two to four relapses, 4% experiencing five or more relapses, and a total relapse rate of 48%. This is a significant number that necessitates more attention and examination from the patients’ own perspectives.

### Study design

A phenomenological research design was employed. This is a reflective and inductive method best suited to revealing how participants interpret their relapse experience^29^.

### Participants and sampling

The participants were purposively selected based on specific criteria relevant to the study objectives. Following Creswell’s^30^ recommendation for phenomenological studies, a sample size of 3–25 participants was considered appropriate to capture diverse experiences. Accordingly, nine adults who had experienced substance use relapse and were able to provide detailed information were recruited with assistance from a center officer.

#### Inclusion criteria

- Adults who had previously relapsed after a substance use disorder.
- Adults who had completed a rehabilitation program, either through formal centers or informal family- and community-based support.

#### Exclusion criteria

- Adults unable to provide informed consent or coherent accounts of their experiences.

Participants were approached through the center officer, and the purposive sampling method was chosen to ensure that those selected could provide rich, relevant insights into relapse experiences.

### Interviewees’ sociodemographic characteristics

The study included nine male participants with a documented history of recurrent substance abuse relapse. The participants were all adults, aged 36–53 years, with a mean age of 44 years. The majority of the participants held a diploma (*n* = 6), followed by a bachelor’s degree (*n* = 2) and high school education (*n* = 1). Slightly more than half were divorced (*n* = 5), whereas the remaining half were unmarried (*n* = 4). In terms of employment, four were self-employed, four worked in the government and private sectors (two each), and one was employed by a nongovernmental organization (NGO).

With respect to substance abuse patterns, six participants reported polysubstance use. The other two engaged in dual-substance use (cigarettes and khat), whereas only three participants reported single-substance dependence (exclusively alcohol, e.g., local beer or araki and beer). Cigarettes and khat were the most commonly consumed substances, each used by six participants, followed by alcohol (*n* = 5) and cannabis (*n* = 2. Furthermore, relapse was measured based on clinical records. For each participant, the frequency of relapse was recorded as the total number of documented recurrences of the condition requiring clinical attention after an initial period of remission. In our sample, the frequency of relapse ranged from 2 to 5 episodes per participant during the follow-up period (Table 1).

**Table 1:** Interviewee background characteristics.

Interviewee	Age	Educational status	Marital status	Previous occupation	Type of substance used	Relapse frequency
01	36	Bachelor degree	Unmarried	Self-employed	Cannabis, Cigarette, Khat for 18 years	3
02	53	Diploma	Divorced	Private (Metal Work)	Alcohol (e.g. Araki or local beer and beer) for 14 years	5
03	45	Diploma	Divorced	Self-employed	Cigarette, Khat	2
04	49	High school	Divorced	Self-employed	Cigarette, Khat	3
05	40	Diploma	Unmarried	NGO	Cigarette, Khat, alcohol	3
06	42	Bachelor degree	Unmarried	Government employed	Alcohol	4
07	50	Diploma	Divorced	Self-employed	Cannabis, Cigarette, Khat	3
08	43	Diploma	Unmarried	Government employed	Alcohol	2
09	37	Diploma	Divorced	Private organization	Alcohol, Cigarette, Khat	5

### Instrument

Semistructured in-depth interviews were employed to elicit interviewees’ experiences, feelings, and views. An interview protocol was prepared by the researchers. Through a panel discussion, the researchers discussed each item and developed a single semi-interview guideline containing the agreed-upon items to measure the phenomenon. The guidelines cover a wide range of issues, including interviewees’ sociodemographic characteristics, substance use incidence, and relapse, how they react to it, and coping perspectives. The interview items were translated into the Amharic language, in which the participants were able to communicate and be administered accordingly.

### Data collection

As one of the coauthors was previously affiliated with the organization, the rehab officers were directly contacted. The participants were recruited and contacted through rehab officers. Seasoned interviewers meticulously gathered the data. To maintain a working relationship and attain an optimum response, the interviewees met two times prior to the interviews and discussed issues other than the research objectives.

Throughout the interview, attention was drawn to and asked about the participants’ nonverbal clues (e.g., their body language, facial emotions, and speaking tempo). The response was elaborated by probing and paraphrasing on the basis of these clues. The interviews took place all in one day. On average, a single interview lasted 30–55 min.

### Data analysis

Data analysis was undertaken in parallel with data collection, with discussion of the emerging themes among the researchers. An interpretive phenomenology analysis (IPA) was employed as it is designed to address research questions that are open, exploratory, and directed primarily at how participants make sense of their experiences^31^. Assuming Finlay’s suggestion, we followed systematic and iterative procedures through adapting the six common data synthesis strategies and steps^32^.

#### Step 1: Reading and rereading

The audio recordings were transcribed verbatim into Microsoft Word documents. The transcripts were read repeatedly until the researchers were fully immersed in the data. The plain texts were then imported into OpenCode (v.4.03). Following data familiarization, initial naming was performed. Responses reflecting participants’ experiences with similar or nearly identical semantic content were identified and labeled using descriptive terms or phrases. Coding was carried out for each relevant statement and phrase to indicate specific patterns of interviewees’ relapse experiences.

#### Step 2: Development of emergent codes

The initial naming labels were transformed into more meaningful codes or phrases. For example, initial labels such as peer influence, lack of control, and powerlessness were synthesized as losses of agency due to peer pressure.

#### Step 3: Abstracting and integrating themes

Related codes were clustered, and overlapping codes were collapsed into broader categories or themes based on conceptual relationships. This was accomplished through a code amalgamation process, and distinctions between categories were refined to reflect participants’ shared experiences. For example, peer encouragement to use substances and exposure to maladaptive advice from patients reflected the broader theme of social environment as a trigger of relapse.

#### Step 4: Move to the next case

We deliberately bracketed the themes extracted from the previous case to maintain openness to the individuality of each new case, avoiding influence from earlier interpretations.

#### Step 5: Searching for patterns across cases

Cross-case analysis began after completing Steps 1–4 for each case. Emergent clustered themes were compared across all cases to identify shared patterns while noting unique or idiosyncratic elements. Superordinate themes were developed by grouping related themes.

#### Step 6: Taking interpretations to deeper levels

This step extended the analysis beyond coding and clustering toward deeper interpretative engagement with the data. Participants’ metaphors, temporal dimensions (how they describe past relapse experiences, present challenges, and future hopes), and relevant theoretical insights were used to enrich the understanding of relapse experiences.

Finally, two independent coders analyzed the data, and panel discussions were held to refine codes, categories, and themes. Each code was reread, similar codes were merged, and commonly recurring themes were identified and centered on participants’ experiences of substance use relapse.

### Quality control

To guarantee data reliability, different verification strategies were employed, including credibility, transferability, dependability, and conformability. Credibility was established via data triangulation, which included interviews, observational data, and investigator triangulation^33,34^, in which two researchers independently reviewed, debated, and cross-verified the findings. Prior to data analysis, we critically examined our assumption about the perspectives of substance abuse relapse, as advised by Priest^35^. Potential biases among researchers were openly acknowledged, reflected, recognized, and intentionally suspended. These include demographic factors, preconceptions, experiential background, and relational dynamics with participants^33^. This was accomplished via a bracketing approach.

Transferability was guaranteed by providing a thorough description of the organization and participants^36,37^. This enables readers to make possible comparisons as they attempt to relate the findings to their own circumstances^33^. To ensure dependability, a clearer explanation of the data collection and analysis processes was provided^33,36,37^, showing the transition from simple classification to more sophisticated coding frameworks.

To improve conformability, respondent validation, also known as member checking^33,34,36,37^ was performed with a subset consisting of one-third of the research participants. Further methodological rigor was enhanced by ensuring transparency in procedures, consulting with seasoned qualitative researchers, and implementing systematic coding protocols.

Negative cases were also considered, along with the investigation of alternate explanations for data that seemed to contradict prevailing trends, as a strategy for enhancing the quality of explanations. When such issues surfaced, we examined them in depth, compared them with emerging themes, and integrated them to provide nuanced interpretations. For example, one participant raised unpleasant emotions (e.g., loneliness) as a relapse trigger, whereas the other focused on avoiding such emotions (e.g., guilt) and staying abstinent in the face of pressure to relapse. The display of unpleasant emotions ensured that both convergent and divergent viewpoints were presented. This could serve as a risk factor as well as an opportunity to desist from relapse. Finally, fair dealing is also ensured by avoiding presenting the opinions of some individuals or circumstances as the only truth regarding any research subject being studied^33^.

## Results

Table 2 presents the main themes and categories identified in the study, along with their definitions and the specific research questions they address. It illustrates how adults in Ethiopia experience relapse, the factors influencing relapse, and effective coping strategies, providing a clear link between the research questions and the qualitative findings.

**Table 2:** Summary of themes, categories, and their alignment with research questions.

Research question	Theme/Category	Definition/Description
1. How do adults in Ethiopia experience relapse?	Peer exposure and indifference	Exposure to peers using substances and lack of personal accountability facilitate initial and repeated substance use.
	Substance use and reuse as a leisure time activity	Using substances to occupy unstructured time or as recreational activity.
2. What factors influence relapse among adults in Ethiopia?	Individual level: Negative emotions	Emotional distress, cravings, and regret trigger relapse.
	Individual level: Loneliness	Feelings of isolation increase vulnerability to substance use.
	Individual level: Lack of self-confidence	Low self-efficacy and fear of social judgment lead to relapse.
	Environmental level: Social environment	Peer influence, family interactions, and social gatherings affect relapse risk.
	Environmental level: Physical environment	Accessibility of substances and exposure to previous substance-use locations trigger relapse.
	Periodic unemployment	Job loss or economic instability increases stress and relapse risk.
3. What coping strategies are effective for adults experiencing relapse in Ethiopia?	Self-motivation and fighting negative emotions	Intrinsic motivation and cognitive strategies help maintain abstinence.
	Modifying the physical environment	Avoiding high-risk locations reduces relapse risk.
	Establishing supportive social systems	Family, peers, and professional support aid recovery.
	Obtaining mental strength and planning for future living	Psychological resilience and structured life planning mitigate relapse.
	Spiritual healings and learning	Engagement in religious or spiritual practices provides cognitive diversion and emotional support.

### Adults relapse experience

On average, participants reported consuming half a pack of cigarettes, 100 g of khat, and seven bottles of beer daily, along with an unspecified quantity of areki (a local alcoholic beverage), until intoxication was reached. Prior to their substance use disorder, participants had been employed in private firms, government institutions, or self-employment but subsequently lost their jobs due to persistent substance abuse and relapse. Frequent relocation between cities occurred as a consequence of unemployment and repeated relapses.

The participants reported experiencing multiple relapse episodes, ranging from a minimum of two to a maximum of six. One participant recounted:

I have had five explicit relapses and one implicit recurrence. I received treatment twice at Amanuel Hospital and three times at a spiritual healing site with holy water (Interviewee 2, September 18, 2024).

### Theme 1: Associations with substance-abusing peers coupled with personal indifference

Two primary factors were identified as contributing to the incidence of substance abuse as well as reuse. The first involved associations with substance-using peers coupled with personal indifference. The participants described scenarios where mere proximity to users without direct coercion predisposed individuals to substance use. Critically, exposure alone did not necessitate consumption; rather, the absence of personal accountability proved to be a determinant. One 36-year-old participant elaborated:

I observed my peers smoking on the way back from the bank to campus. Without due consideration, I requested a cigarette from one friend. Upon receiving it, I inhaled once and immediately experienced its effects (Interviewee 1, September 18, 2024).

A 53-year-old participant reported initiating consumption of local alcoholic beverages (areki) and beer when relocating for employment purposes. Initial substance use experiences are consistently characterized by adverse physiological reactions (e.g., coughing) and negative affective reactions (e.g., disgust). One participant noted that “during my initial cigarette use, I experienced immediate aversion, including feelings of disgust and coughing. However, prolonged exposure within my peer group where cigarettes were routinely shared led to gradual desensitization and eventual development of smoking attraction” (Interviewee 1, September 18, 2024).

The findings demonstrate that first substance use typically elicits adverse physiological and affective responses, including disgust and coughing. However, repeated exposure to substance-conducive environments appears to facilitate habituation and subsequent positive reinforcement of addictive behaviors. Notably, as aversive reactions diminish, favorable associations with substance use emerge.

### Theme 2: substance use and reuse as a leisure time activity

A second mechanism to relapse patterns involves leisure time utilization. The participants reported frequently engaging in substance use and reuse as a means of occupying unstructured time. For example, one participant noted, “I would chew [khat] during winter evenings after returning from campus” (Interviewee 1, September 18, 2024). This pattern suggests that substance use may function as both a recreational activity and a temporal coping mechanism among these participants.

### Factors influencing relapse

#### Theme 1: Individual level

##### Category 1: Negative emotions

Negative emotional states emerged as a significant precipitating factor in substance use relapse among participants. Affective disturbances substantially compromised individuals’ capacity to maintain abstinence from previously discontinued substance use patterns. They reported experiencing vivid sensory hallucinations (olfactory, gustatory, and tactile) from substances during abstinence periods. These craving manifestations frequently precipitate relapse episodes, as described below: “I feel the substance’s presence through smell, taste, and physical sensation despite its absence. When these symptoms occur, I procure and consume them covertly” (Interviewee 1, Sep 18, 2024). However, postconsumption typically involves “acute regret and feelings of badness, then I use substances again” (Interviewee 1, Sep 18, 2024), establishing a cyclical pattern where subsequent substance use functions as an avoidance mechanism for these negative emotional states. This phenomenon aligns with contemporary models of addiction that characterize relapse as both craving-driven and negatively reinforced through affect regulation.

In this case, emotional triggers for relapse manifest in various ways. The participants reported experiencing psychological cravings for substances even during abstinence, which undermined their resolve to remain sober. These cravings often lead to covert substance use as a means of coping with intrusive thoughts and emotional distress. Subsequently, feelings of guilt or regret following use frequently perpetuated the cycle of relapse. These findings illustrate a reciprocal link between emotions and substance use where sensory cues (e.g., smelling cigarettes) may trigger relapse, and subsequent negative affect (e.g., guilt) reinforces further use.

An additional factor associated with emotional experience is ‘existential regret regarding one’s life path.’ One participant expressed this as.

I felt profound regret over the loss of my girlfriend, friendships, and employment. My family and neighbors expected me to maintain a respectable appearance, secure stable employment, build a family, and have children - yet I failed to meet these societal expectations. During my holy water treatment at Shinquru Michael [a church located in Entto Mountain, north of Addis Ababa], these thoughts overwhelmed me, causing deep distress. In response, I relocated to a nearby small shop, engaged in secretive smoking, and eventually returned to the monastery (Interviewee 1, September 18, 2024).

The emergence of negative affective states, particularly feelings of regret or perceived loss, constitutes a significant relapse risk factor. This dynamic is illustrated in the following narrative:

I feel deep failure, stemming from both romantic and occupational instability, which has contributed to my renewed substance use. These feelings are compounded by my inability to establish marital or familial bonds and by my continued financial dependence on my siblings (*Interviewee 1*,* Sep 18*,* 2024*).

An additional source of negative affect stems from processes of social comparison.

Upon observing peers who have achieved significant financial advancement, as evidenced by home ownership and vehicle acquisition, I experience intense feelings of regret and frustration regarding my own perceived lack of socioeconomic progress. These affective states frequently precipitate relapse into alcohol use as a maladaptive coping strategy, resulting in dissociative behaviors and increased social disinhibition (Interviewee 2, September 18, 2024).

##### Category 2: Loneliness

Loneliness represents another emotion-driven factor contributing to relapse, particularly when stemming from life stressors (e.g., marital dissolution). The participants reported:

After returning from work to the residence I had previously shared with my spouse, I experienced profound loneliness. This affective state frequently precipitates social-seeking behaviors, including visiting drinking establishments to consume alcohol. I attribute my increased alcohol consumption directly to the absence of my partner (Interviewee 2, September 18, 2024).

The experience of loneliness subsequently engendered feelings of hopelessness, which the participants reported managing through alcohol consumption. He articulated this psychological state as follows: “Despite being gainfully employed, I perceive my earnings as meaningless owing to my solitary existence. I frequently question the purpose of my labor, as I lack a spouse or family for whom to provide” (Interviewee 2, September 18, 2024).

The participants reported reusing alcohol as both an emotional escape mechanism and a social facilitation strategy. The communal drinking environment serves multiple psychological functions: alleviating loneliness, generating positive affect, and enabling unrestricted social interaction. It is described by the participant as.

Social drinking at local establishments provides a sense of liberation and emotional catharsis. These spaces allow uninhibited self-expression, ranging from casual conversation to emotional outbursts, which contributes significantly to subjective well-being. My typical pattern involves moderate consumption while purchasing drinks for peers, reinforcing social bonds (Interviewee 2, September 18, 2024).

##### Category 3: Lack of self-confidence

Deficits in self-efficacy represent another significant barrier to sustained recovery. Individuals frequently report anxiety about social rejection, stigmatization, and stress from their former substance-using networks, which often precipitates relapse. A participant articulated that “Despite maintaining abstinence for several weeks, I ultimately resumed substance use due to anticipatory shame and fear of stigmatization by peers in the consumption environment” (Interviewee 1, September 18, 2024).

This phenomenon does not necessarily reflect actual social labeling or stigmatization but rather stems from internalized beliefs, self-generated affective states, and cognitive justifications. An additional psychological factor exacerbating self-efficacy deficits involves impaired volitional control, manifested through inconsistent commitment and failure to sustain abstinence. The participants reported significant difficulties (linked to cognitive dissonance) in maintaining behavioral consistency regarding substance avoidance, as exemplified in the following accounts.

Considering my sister’s significant sacrifices including enduring emotional distress, providing financial support, and even redirecting resources from her own children to me, I decided to refrain from substance use permanently. Despite this commitment, I found it difficult to sustain consistent progress in my recovery efforts (Interviewee 8, September 18, 2024).

#### Theme 2: Environmental level: Social and physical

##### Category 1: Social environment

The social environment plays a critical role in either facilitating recovery or precipitating relapse. An individual resolve to maintain sobriety often proves inadequate without concurrent support from one’s social network, including family members, peers, and other significant relationships. This phenomenon is exemplified in the following participant account:

Following two months of sustained recovery, I attended wedding ceremonies where the bride groom was my best. Sociocultural pressures within that context led to my renewed substance use, despite my prior commitment to recovery (Interviewee 4, September 18, 2024).

Similarly, another participant described his relapse experience following substance use treatment:

After successfully completing a 40-day rehabilitation program during the COVID-19 pandemic in 2019, I was discharged with the requirement to attend monthly medication reviews and group therapy sessions, which I maintained for six months. However, in late 2019, I resumed consuming beer and Areki at social gatherings, leading to an explicit relapse that necessitated admission to the current hospital (Interviewee 8, Sep 18, 2024).

Social influences often operate without conscious awareness, as significant others may inadvertently contribute to relapse behaviors despite positive intentions. While ceremonial participation can serve as a protective factor, it simultaneously presents risks through peer pressure mechanisms. This paradoxical dynamic is exemplified in one participant’s description:

Peers actively encouraged me through persistent persuasion, framing alcohol consumption as a therapeutic alternative to depressive states rather than recognizing its maladaptive nature (Interviewee 4, Sep 18, 2024).

Peer influence exhibits situational variability, with heightened susceptibility occurring during deviations from routine activities. This phenomenon is illustrated in the following participant narrative:

While awaiting my partner outside a beauty salon, I received unsolicited encouragement from a friend to consume alcohol as a leisure activity, despite awareness of my partner’s objections. This resulted in progressive consumption, culminating in observable intoxication (Interviewee 6, Sep 18, 2024).

Another very similar but different challenge is detrimental advice and information from patients in the hospital.

There are individuals who smoke cigarettes illegally here in the rehab center and insist on tobacco use. They even give me information, as I can bring beers if I have money at hand (Interviewee 2, Sep 18, 2024).

A distinct yet related concern involves exposure to maladaptive advice and misinformation from peers within treatment facilities. Some individuals actively engage in tobacco use during rehabilitation and encourage similar behaviors among fellow patients. As one participant reported,

Certain patients within the rehabilitation center continue to smoke and encourage tobacco use. Moreover, they have suggested that purchasing and consuming beer would be possible if financial resources were available (Interviewee 2, Sep 18, 2024).

##### Category 2: Physical environment

The physical environment, particularly substance availability, substantially influences susceptibility to relapse. The participants reported maintaining abstinence for extended periods through various support systems (familial, intrinsic motivation, and spiritual interventions). However, re-exposure to their original residential environments frequently results in relapse. This pattern is expressed by a participant as.

Having achieved two months of sustained recovery in a monastic setting, where I developed considerable psychological resilience, I consulted with my family before returning home. Upon returning to my previous residence, I immediately resumed substance use (Interviewee 5, Sep 18, 2024).

The participants’ familiarity with specific geographical locations associated with prior substance procurement and consumption elicited powerful cognitive-emotional recall. These environmental cues activate affective responses that subsequently manifest as renewed substance use behaviors (e.g., chewing and smoking). The accessibility of substances within one’s immediate treatment surroundings was found to substantially compromise relapse prevention efforts. A participant described the following.

Despite multiple engagements with spiritual interventions at sacred sites, I experienced only brief periods of non-use. The close proximity of these treatment locations to establishments where substances were accessible created a cyclical pattern of recovery and relapse, significantly impeding my rehabilitation progress (Interviewee 9, Sep 18, 2024).

#### Theme 3: Periodic unemployment

Periodic unemployment, particularly among private sector and self-employed individuals, emerges as a significant risk factor for relapse. The structural vulnerability created by work discontinuity appears to facilitate renewed substance use patterns. This phenomenon is evidenced in the following participant account:

During extended periods of occupational inactivity (e.g., termination from a car reassembling company), I consistently experienced negative affective states accompanied by alcohol cravings. This progression from intermittent drinking to habitual use ultimately resulted in a full relapse (Interviewee 2, Sep 18, 2024).

### Coping strategies to quit relapse

#### Theme 1: Self-motivation and fighting negative emotions

The findings suggest that durable restraint is most effectively achieved when predicated on intrinsic motivation, as evidenced by a participant: “Initial cessation attempts were primarily self-motivated.” Mitigation-related psychological distress indicators appear crucial for maintaining long-term recovery. One participant noted that “while intermittent cognitive avoidance and recognizing it were used, these strategies remained important  .” Another participant described his utilization of intellectual engagement through cognitive diversion strategies as a relapse prevention mechanism:

Throughout the rehabilitation process, I read theological scriptures as a cognitive distraction from substance-related thoughts. Complementary exposure to historiographical works served the dual purposes of intellectual enrichment and emotional regulation. These deliberate practices significantly aided my navigation of recovery challenges (Interviewee 7, Sep 18, 2024).

When individuals experience accumulations of distressing affective states, effective coping necessitates the strategic redirection of attention away from maladaptive emotional processing. This can be operationalized through several evidence-based approaches: (1) engagement in alternative activities that promote cognitive absorption, (2) environmental modification to avoid geographic triggers associated with negative emotional conditioning, and (3) proactive positioning in contexts that minimize exposure to emotionally provocative stimuli. Such diversionary tactics function as psychological buffers, interrupting the cyclical reinforcement of deleterious emotional patterns that frequently precipitate relapse behaviors.

#### Theme 2: Environment: Physical and social

##### Category 1: Modifying the physical environment

The participants emphasized the critical role of changing residences or physical environments in supporting sustained recovery. Strategic avoidance of high-risk geographic locations associated with prior substance use emerged as a key protective factor. As one participant articulated,

Effective relapse prevention necessitates deliberate avoidance of establishments where alcohol is commercially available. Additionally, it is important to avoid social gatherings that present environmental triggers (Interviewee 9, Sep 18, 2024).

While environmental modification (including both physical and social ) may not constitute a comprehensive strategy, geographic relocation can facilitate recovery by severing associations with substance-related cues. This approach operates through physical separation from locations linked to prior substance use, social disengagement from substance-using networks, and immersion in novel environments that foster alternative cognitive associations. Such measures aim to lessen conditioned responses and prevent the activation of substance-related memories, potentially facilitating both short-term and long-term recovery. However, as evidenced by one participant’s account, this strategy alone may prove insufficient:

Following discharge from a rehabilitation center, I relocated to Shashemenie, Hawassa, and Chiko towns, concurrently terminating contact with substance-using associates, which allowed only limited sustained recovery. Despite these measures, I experienced both treatment nonresponse and dosage escalation, progressing from 100 to 200 g to more than 300 g of khat daily, accompanied by polysubstance use involving cannabis (Interviewee 1, Sep 18, 2024).

Another participant similarly emphasized the strategy of “avoiding returning to places where alcohol is serving.” However, collective participant accounts suggest that environmental modification alone proves inadequate as a relapse prevention mechanism. More effective outcomes appear contingent upon the interplay of multiple factors, including sustained personal commitment and restricted substance availability in one’s surroundings. The data indicate that participants often lack the necessary psychological resilience to maintain self-restraint, compounded by the additional challenge of residing in areas characterized by widespread khat cultivation and distribution networks.

##### Category 2: Establishing supportive social support systems

The establishment of functional social support systems, particularly those involving significant others, is a critical protective factor against relapse. Positive interpersonal environments encompassing familial, peer, and community networks exert substantial influence on long-term recovery outcomes. A participant shared as.

The sustained recovery I experienced was facilitated by guidance from both impartial and affiliated professionals in the field (Interviewee 9, Sep 18, 2024).

A participant corroborated this perspective, stating:

During my recovery, a childhood acquaintance demonstrated empathic understanding by consistently advising me against frequenting establishments serving Areki while proactively monitoring my adherence to this behavioral modification. This individual also instituted alternative socialization patterns, specifically transitioning our interactions to café environments. Concurrently, immediate family members provided substantial support: my sister facilitated treatment access by coordinating placement in a rehabilitation center, and my cohabiting brother offered ongoing domestic reinforcement. Extended family members, including cousins, contributed to my recovery by fostering alternative cognitive engagements, particularly encouraging an interest in literary pursuits (e.g., reading books) (Interviewee 2, Sep 18, 2024).

The participants emphasized the critical importance of deliberate social network building or peer selection in maintaining recovery. Participant five articulated, *“*A fundamental recommendation I would propose involves the judicious selection of peer associations*—*specifically prioritizing individuals who exert neither direct nor indirect influence toward substance use behaviors” (Sep 18, 2024).

#### Theme 3: Obtaining mental strength and planning for future living

The cultivation of psychological resilience and strategic life planning was found to be a vital factor in mitigating relapse. This encompasses both cognitive commitment and behavioral adherence to self-restraint principles. A participant noted the following:

The primary attribute of psychological fortitude was divine support, followed by maximal personal commitment. Subsequently, collaborative planning with familial support systems facilitated posttreatment occupational stabilization (Interviewee 1, Sep 18, 2024).

The integration of spiritual reliance, specifically through divine trust and reinforced abstinence conviction, serves as a critical psychological resource that enhances recovery perseverance. Furthermore, the resolution of occupational instability through familial intervention (as exemplified by the participant’s brother securing employment) constitutes an important protective factor that reinforces long-term relapse prevention through structured daily engagement and socioeconomic stabilization.

#### Theme 4: Spiritual healings and learning

The utilization of spiritual interventions, including engagement with religious institutions (e.g., churches, mosques, monasteries, and holy water), has emerged as a salient way to cope with relapse. This approach is illustrated in the participant’s account as follows:

During periods of heightened relapse vulnerability, I actively sought religious sanctuary through church attendance, engaging in prayer and exposure to sacred choral music (madrigals and canticles). These practices facilitated temporary cognitive diversion from substance-related ideation, subsequently reinforcing a pattern of repeated religious participation (2–3 times weekly). The complementary strategies included devotional text study (biblical scriptures) and reading of historiographical literature, supplemented by occasional visits to secular recreational venues (Interviewee 9, September 18, 2024).

## Discussion

### Experiencing relapse

Despite receiving therapies on the basis of friendships, family support, religious interventions, and hospitalization, the results revealed that participants had a history of multiple relapses up to five times. These findings align with earlier studies . For instance, McQuaid, et al.^38^ documented six or more relapses. Similarly, five or more relapses were discovered by Abegaz^3^ and Sohrabpour, et al.^11^.

Participants relapse when they are around friends who take substances and use them for amusement. On the other hand, indifference or casual experimentation are seen to be more important factors in the initiation of substance use as well as relapse than mere exposure to friends who use substances. While people may first have adverse reactions when they try narcotics, they are eventually attracted to it and become active users.

A major influence on the microsystem was the presence of friends who were substance abusers. The social dynamics in these connections fostered a culture that normalized substance experimentation. An increase in substance use or relapse was caused by peer pressure and the need for social acceptance within their close social circles ^39^. Although experimentation initially elicits adverse feelings, people eventually become regular users due to the appeal of substance usage.

The findings revealed that despite undergoing various treatments (e.g., family-based, friendship-based, religious-based, and hospitalization), participants continued to relapse. This suggests that the larger social environment (exosystem), including the availability of substances and the normalization of substance use within their community, plays a critical role in their ability to maintain recovery^25,40^.

### Factors causing relapse

#### Individual-level emotional factors

At the individual level, emotional reasons such as regret and loss are significant triggers for relapse. Individuals are often emotionally drawn to substances, even in their absence. Feelings of regret, often stemming from difficulty in adapting to life changes and unfavorable self-comparisons, can influence an individual’s intention to return to substance use as a coping mechanism. Loneliness can further intensify regret and hopelessness, reinforcing the cycle of relapse. These emotional dynamics were identified as key contributors to relapse among our participants. This finding is in line with those of previous works. Psychological distress^39^ and willingness and pleasure^41^ are linked to relapse; approximately 90% of relapse cases are driven by emotion-based factors, with individuals struggling to manage negative emotions without substances^20^. Adults relapse as a tactic to counter adverse emotions, stay occupied, cope with loneliness^42^, and seek solace in social connections formed in substance use environments, where they experience positive emotions such as happiness and enjoyment, often using drugs as a means to escape emotional turmoil^43,44^. Additionally, the disease model suggests that relapse risk is strongly influenced by genetic, lifestyle, and familial factors, whereas the adaptive perspective highlights individual differences, such as in coping strategies and stress management^45–47^.

Low self-confidence also contributes to relapse. Individuals with poor self-confidence often struggle to assert themselves, say no, or resist pressure from others. This is frequently tied to fear of rejection, for example, concerns that refusing substances may lead to social exclusion or labeling. Although actual rejection may not occur, participants often justified their relapse through these fears. A lack of self-control further compounds this challenge, as individuals with low confidence or efficacy are less likely to maintain the self-discipline and decision-making required for long-term recovery^26,27,43,48^. The belief that refusing substance use may result in social rejection strengthens their engagement in consuming khat or alcohol. The findings also align with the role of the microsystem, particularly the influence of friends and social networks^11^. Vulnerability to peer influence underscores the interaction between personal psychological factors and social dynamics. The participants indicated that loneliness and the need for social connection drove them toward substance reuse. They frequently sought friendships in settings where substances were consumed, as these environments provided an escape from negative emotions. Such dynamics within immediate social circles can worsen emotional difficulties and ultimately contribute to relapse.

### The immediate environment: Social-cultural and physical

The immediate environment and communication among individuals, including social, cultural, and physical settings, play crucial roles in facilitating relapse among adults with substance use disorders. This encompasses the influence of significant others (e.g., family, friends, and community members), cultural practices (e.g., norm of chewing khat), and the physical environment (e.g., availability and production). Social and cultural dynamics may encourage individuals to reinitiate substance use. While there may not be an intentional effort to harm, these environments foster behaviors that promote enjoyment, social harmony, and relationship formation. Societal influence and cultural expectations, especially during events such as ceremonies, play a significant role in shaping relapse. These findings are consistent with earlier studies. For example, relapse is reinforced by sociocultural and contextual factors (e.g., encouragement from friends, family members, and acquaintances who are addicted) and is further increased by substance accessibility^39,41,42^.

This finding is also consistent with different theoretical perspectives. For example, bioecological theory underscores the impact of microsystems, which encompass individuals’ immediate environments and social circles that influence adult behavior. These influences can be negative, promoting behaviors such as smoking, chewing, and alcohol consumption. The macrosystems within the theory are linked to cultural values and societal beliefs regarding substance use prevalent in a community. These shared norms and expectations influence adults’ behaviors, particularly substance consumption^40,49^. This was exemplified by participants’ experiences in a place recognized for its khat plantation, where khat chewing is culturally practiced and regarded as a traditional activity.

#### Unemployment and economic factors

Periodic unemployment constitutes another factor contributing to the resurgence of substance abuse among adults. This is common among nongovernmental employees as our participants. Inability to secure employment creates increased free time, which may lead to a higher propensity for recurrence. Adults with a history of substance abuse may cease substance use while engaging in daily routines; however, nonstructural unemployment often results in relapse, depending on the severity of the addiction. Severe substance abuse behavior can also lead to workplace challenges, such as tardiness, poor performance, failure to meet deadlines, and conflicts with colleagues or supervisors, ultimately resulting in job loss, a phenomenon referred to by Benjamin^45^ as “occupational disputes.” Therefore, temporary loss of employment was a significant trigger for relapse among participants.

According to Bronfenbrenner’s bioecological theory, the association between intermittent unemployment and relapse can be understood at the microsystem, mesosystem, and exosystem levels. Within the microsystem, unemployment may cause heightened stress and social isolation. Unemployed adults often experience a lack of social support or meaningful connections, driving them toward substance use as a coping mechanism. Immediate social environments, including peers who engage in substance use, can reinforce these behaviors, establishing a difficult-to-break cycle, as evidenced by participants reporting substance use, specifically with khat and alcohol.

The exosystem, referring to broader social structures that indirectly influence the individual (e.g., community resources, economic conditions, and institutional policies, particularly within workplaces), also plays a role. Periodic unemployment reflects broader economic instability and limited availability of job opportunities. Participants’ perceptions of limited job prospects or job loss, often linked to relapse, may cultivate feelings of hopelessness, leading to substance use as an escape. The presence or absence of supportive employment programs and community initiatives can either alleviate or exacerbate the impact of unemployment on substance use. Furthermore, difficult living conditions, such as unemployment and financial strain, are linked to a greater chance of relapse, which is consistent with Bandura’s^40,41^ social learning theory and Lazarus and Folkman’s^50^ stress and coping theories.

### Quitting relapse

#### Individual-Level: Self-Motivation and emotional regulation

The participants’ accounts highlight the critical role of self-motivation in preventing relapse. Self-motivation is essential for individuals to effectively avoid the recurrence of substance use. These insights suggest that self-motivation, alongside the management of negative emotional states, corresponds to individual-level factors within bioecological systems theory^40,49^.

Engaging in activities such as reading books and contemplating the consequences of relapse can help individuals manage negative emotions by redirecting their focus away from thoughts linked to substance use. The capacity to regulate these emotions while maintaining motivation is fundamental to achieving successful continued recovery.

#### Environment

##### Relocating to a new physical environment

Relocating to a new physical setting assisted participants in separating themselves from substance use triggers (e.g., particular persons, locations, or memories). As a result, fewer substance-related cues were presented, and healthier routines were easier to establish. They might, however, also encounter challenges such as the possibility that they would still be exposed to substances in their new setting. For example, participants who lacked strong personal resilience reported difficulties sustaining their recovery. These finding illustrate the potential benefits and practical limitations of environmental programs, emphasizing the need for individual commitment and societal support to ensure successful implementation. This process is consistent with the exosystem and macrosystem levels of ecological systems theory, which accentuate the impact of larger social and environmental factors on individual behavior^40,49^.

##### Supportive social environment (significant others)

The presence of a nurturing social environment substantially reinforces an individual’s commitment to recovery, as demonstrated by participants’ interactions with family and friends. They indicated the importance of empathetic friends who promote positive behaviors, discourage relapse, and provide guidance in avoiding risky environments, such as advising them to spend time in cafes instead. These experiences reflect the significance of the microsystem and mesosystem within Bronfenbrenner’s ecological theory, where immediate environments and close relationships enhance coping capacity and resilience against relapse^40,49^. This is also consistent with social control theory, which posits that strong bonds with family, friends, work, religion, and other aspects of society motivate individuals to engage in responsible behavior and refrain from relapse^51^.

Building on these findings, families and social networks can be utilized more actively in the relapse prevention process. For example, educational programs can increase families’ knowledge of relapse triggers and supportive communication strategies. Families can also be equipped with practical strategies to encourage safe social engagement, promote constructive routines, and provide consistent emotional support. Such interventions strengthen the capacity of social networks to act as active resources and sustain long-term recovery.

#### Obtaining mental strength and planning for future living

Psychological preparedness is an additional crucial factor for supporting continued recovery. The participants highlighted that maintaining personal commitment and developing future plans are essential for preventing relapse. This concept underscores the importance of individual-level factors as well as the chronosystem level within Bronfenbrenner’s ecological framework. The capacity to envision a positive future and sustain psychological resilience is vital for long-term recovery^40,49^.

#### Spiritual healings and learning

Spiritual practices and readings emerged as key relapse coping strategies among participants. These principles were put into reality through tangible activities such as attending churches or mosques, praying, and reading scriptures (e.g., the Bible or the Quran). These activities provided them with emotional support and fostered a sense of community. The participants turned to these places of worship for comfort during periods of relapse, which reflected the ecological theory of Bronfenbrenner at the macrosystem level^40,49^. This means that preaching emphasizes personal perseverance in the face of hardship and that promoting responsibility may drive people to refrain . Spirituality supported rehabilitation attempts by maintaining emotional relief, increasing personal resilience and fostering the growth of supportive networks. However, inconsistencies in spiritual commitment were also noted.

Overall, relapse is strongly influenced by the intricate interactions among the different levels of ecological theory. After leaving the rehab center or hospital, for example, participants may rely on family support (microsystem); however, if they encounter emotional distress, such as at work (exosystem), they may relapse. However, these challenges can be mitigated by a combination of family-therapist collaboration (mesosystem) and strong religious values and authority engagements (macrosystem). Such support can strengthen resilience and mitigate personal vulnerabilities (e.g., guilt, loneliness, low self-confidence, etc.) throughout time (chronosystems), increasing the duration of recovery. Furthermore, the findings underscore the relevance of the biopsychosocial model, suggesting that relapse is shaped by the dynamic interaction of biological vulnerabilities, psychological processes, and social determinants, thereby reinforcing the need for integrated theoretical frameworks in understanding substance use disorders.

### Biopsychosocial consequences of relapse, mitigation strategies, and supporting evidence

Findings from the present study illustrate relapse among adults in Ethiopia as a recurrent and multifaceted process shaped by interacting biological, psychological, and social factors. Participants reported experiencing multiple relapse episodes (ranging from two to six), often following brief periods of abstinence achieved through medical, spiritual, or psychosocial interventions. These repeated relapse experiences highlight that relapse is not a singular failure, but rather a chronic and cyclical phenomenon embedded within broader life circumstances.

At the biological and psychological interface, participants’ narratives revealed intense craving experiences characterized by vivid sensory imagery (e.g., olfactory and gustatory sensations), emotional distress, and impaired self-regulation. These findings align with neurobiological models of addiction, which emphasize substance-induced neuroadaptations in reward, stress, and inhibitory control systems that heighten vulnerability to relapse, particularly under emotional strain^21^. In the present study, relapse was frequently triggered by negative affective states such as regret, loneliness, perceived failure, and social comparison, supporting theoretical models that conceptualize relapse as a negatively reinforced coping response aimed at alleviating emotional distress^22^.

Psychologically, participants demonstrated diminished self-efficacy, internalized stigma, and fear of social judgment, which undermined sustained abstinence even in the absence of overt social pressure. The findings suggest that relapse was often driven not by direct coercion, but by internal cognitive justifications and emotional vulnerability. This supports relapse prevention theory, which posits that inadequate coping skills, low confidence, and maladaptive cognitive appraisals increase relapse risk when individuals encounter high-risk situations^22^. The cyclical pattern observed - craving, covert substance use, regret, and renewed use - illustrates how psychological processes perpetuate relapse over time.

Social and environmental factors emerged as central determinants of relapse in this study. Participants’ accounts consistently emphasized the influence of substance-using peers, social gatherings, and culturally normative substance use contexts (e.g., weddings, leisure activities). Importantly, relapse can frequently occurred despite personal motivation to remain abstinent, indicating that individual resolve alone was insufficient without supportive social structures. These findings align with recovery capital theory, which underscores the importance of social support, peer networks, and community engagement in sustaining recovery^23,52^. Additionally, exposure to maladaptive peer behaviors within treatment settings highlights a structural vulnerability that may undermine recovery efforts.

Physical environments also played a decisive role in relapse patterns. Participants described strong conditioned responses to familiar locations associated with prior substance use, with re-exposure frequently precipitating immediate relapse. This finding supports cue-reactivity and conditioning models of addiction, which posit that environmental cues can activate craving and automatic substance-seeking behaviors even after periods of avoidance ^21^. In contexts where substance availability is widespread, environmental modification alone proved insufficient, reinforcing the need for integrated strategies that address both individual vulnerability and structural exposure.

Periodic unemployment further compounded relapse vulnerability by intensifying stress, hopelessness, and unstructured time. The present findings demonstrate how economic instability interacts with emotional distress to increase relapse risk, reinforcing chronic disease models of addiction that emphasize the role of social determinants of health in recovery trajectories^53^.

With respect to mitigation strategies, participants identified multiple coping mechanisms that contributed to periods of sustained abstinence. Intrinsic motivation, cognitive diversion, emotional regulation strategies, and future-oriented planning were central psychological resources that helped interrupt relapse cycles. These findings resonate with evidence supporting cognitive-behavioral and mindfulness-based relapse prevention approaches, which enhance emotional awareness, coping capacity, and self-regulation^24^. Environmental modification, including avoiding high-risk locations and relocating geographically, offered short-term protection but was most effective when combined with psychological resilience and social support.

Social support systems particularly family involvement, peer monitoring, and professional guidance emerged as critical protective factors in maintaining recovery. Participants’ narratives illustrate how supportive relationships facilitated accountability, alternative socialization, and access to treatment, consistent with prior research emphasizing the role of recovery-oriented systems of care^23,53^.

Spiritual practices also played a significant role in coping with relapse, functioning as sources of emotional regulation, cognitive adjustment, and meaning-making. While spiritual healing alone did not ensure sustained non-use, its integration with psychological and social supports enhanced resilience and motivation, highlighting its contextual relevance within the Ethiopian sociocultural setting.

Overall, the present findings provide empirical support for biopsychosocial and chronic disease models of relapse. The findings extend existing theory by demonstrating how relapse frequency is shaped by the dynamic interaction of emotional vulnerability, environmental exposure, social context, and structural instability. These underscore the theoretical and practical necessity of long-term, integrated interventions that move beyond individual-level explanations to address the broader psychosocial and environmental conditions influencing relapse.

## Limitations

Although qualitative research traditionally emphasizes depth of understanding over generalizability, the current study’s limited sample size may constrain comprehensive exploration of the phenomenon under investigation. A more extensive sample could yield additional perspectives and thematic variations concerning substance abuse trajectories, relapse experiences, and coping strategies. Subsequent investigations would benefit from employing larger, more heterogeneous samples combined with methodological triangulation to strengthen both the validity and transferability of findings.

Moreover, throughout the data collection phase, participants may have encountered memory bias, complicating the recall of past experiences. During data analysis, limits may stem from interpretative subjectivity and the management of data bulk, potentially introducing bias and rendering coding and theme creation laborious.

## Conclusion and implications

Participant narratives indicate that substance use behaviors typically originate during youth, motivated by both impulsive decision-making patterns and the utilization of substances as a temporal coping mechanism. Critical developmental periods, particularly adolescence and phases of unemployment, emerge as vulnerable intervals for initiation. Association with substance-using peers does not inherently precipitate problematic substance use or reuse; rather, it is the concomitant attitude of indifference that appears to significantly influence active substance abuse patterns.

Relapse is influenced by a multifactorial etiology encompassing both individual and environmental determinants. At the individual level, psychological factors, including affective states (e.g., regret, anger, loneliness), deficits in self-efficacy, impaired impulse regulation, and attitudinal indifference, constitute significant risk factors. Environmental precipitants include sociocontextual influences such as peer pressure and social settings that facilitate substance use and relapse initiation, as well as physical determinants such as the accessibility of substances within one’s residential environment.

Adults experiencing substance abuse relapse employ diverse coping mechanisms, encompassing both individual-level strategies and those utilizing social, spiritual, and physical environmental resources. The dynamic interaction between intrapersonal characteristics and multilevel environmental factors, including temporal contextual influences, impacts relapse. Considering these complex interactions is vital when holistic intervention approaches for substance abuse recovery are formulated.

Building upon these findings, interventions should adopt a multisystemic approach that simultaneously targets individual-level determinants and contextual environmental influences. By addressing the identified etiological factors of relapse, stakeholders can cultivate more supportive recovery ecosystems and mitigate relapse risk more effectively. Relapse are driven by interconnected biological, psychological, and social factors, underscoring the need for an integrated and evidence-based interventions. Combining medication-assisted treatment with psychological therapies and social support, alongside strengthened recovery-oriented systems of care, can reduce relapse risk and promote sustained recovery. These findings highlight the importance of long-term, holistic approaches in both clinical practice and policy development.

## Data Availability

The interview transcripts are available upon reasonable request from the corresponding author.
